# FAMILY COHESION MODERATES THE RELATIONSHIP BETWEEN ACCULTURATION AND HEALTH AMONG OLDER CHINESE AMERICANS

**DOI:** 10.1093/geroni/igz038.2346

**Published:** 2019-11-08

**Authors:** Kaipeng Wang, Anao Zhang, Fei Sun, Rita X Hu

**Affiliations:** 1 Texas State University, San Marcos, Texas, United States; 2 University of Michigan, Ann Arbor, Michigan, United States; 3 Michigan State University, East Lansing, Michigan, United States; 4 University of Michigan, Ann Arbor, Ann Arbor, Michigan, United States

## Abstract

Migration and resettlement are major life events that affect immigrants’ functioning and health status. Previous research has well-established the influence of acculturation and family cohesion on Chinese Americans’ mental health and health behavior; however, the moderation effect of family cohesion on the relationship between acculturation and self-rated health – a robust measure of an individual’s general health – has not been examined among this population. The purpose of this study is to examine the association between family cohesion, acculturation, and self-rated health among older Chinese Americans. Data came from a survey of 385 Chinese Americans aged 55 and older living in a large metropolitan area in Southwest America through face-to-face interviews. We used logistic regression to examine the association between acculturation, family cohesion, and self-reported health. In general, acculturation was significantly associated with higher odds of reporting excellent or good health after adjusting for demographic and psychosocial covariates; however, the association between acculturation and self-reported health differed by family cohesion. We found that acculturation was positively associated with self-reported health only among those with medium or high family cohesion, but not among those with low family cohesion. Findings highlighted the significance of involving family members and strengthening family support for providing acculturation services, such as language class and healthy literacy education, to older Americans. Family cohesion needs to be considered by health and mental health care providers for older Chinese Americans to further understand the resources and barriers that influence their health service use and health behaviors.

